# Rapid Study on Mefloquine Hydrochloride Complexation with Hydroxypropyl-β-Cyclodextrin and Randomly Methylated β-Cyclodextrin: Phase Diagrams, Nuclear Magnetic Resonance Analysis, and Stability Assessment

**DOI:** 10.3390/pharmaceutics15122794

**Published:** 2023-12-18

**Authors:** Amaury Durand, David Mathiron, Sébastien Rigaud, Florence Djedaini-Pilard, Frédéric Marçon

**Affiliations:** 1 AGIR UR 4294, UFR de Pharmacie, Université de Picardie Jules Verne, 80037 Amiens, France; a.durand@ch-boulogne.fr; 2 Pharmacie à Usage Intérieur, Centre Hospitalier Universitaire d’Amiens–Picardie, 80054 Amiens, France; 3 Plateforme Analytique, Université de Picardie Jules Verne, 80039 Amiens, France; david.mathiron@u-picardie.fr; 4 LG2A UR 7378, UFR des Sciences, Université de Picardie Jules Verne, 80039 Amiens, France; sebastien.rigaud@u-picardie.fr (S.R.); florence.pilard@u-picardie.fr (F.D.-P.)

**Keywords:** mefloquine, cyclodextrins, inclusion complexes, nuclear magnetic resonance spectroscopy, stereoisomerism, drug stability

## Abstract

This study investigates the complexation of mefloquine hydrochloride by cyclodextrins to improve its solubility in order to design an oral solution. This approach may enhance the effectiveness of mefloquine, a drug which can be used for malaria prophylaxis and treatment in children. Mefloquine hydrochloride’s solubility was assessed in different buffer solutions, and its quantification was achieved through high-performance liquid chromatography. The complexation efficiency with cyclodextrins was evaluated, and nuclear magnetic resonance (NMR) methods were employed to determine the interactions between mefloquine and cyclodextrins. Mefloquine’s solubility increased when combined with hydroxypropyl-β-cyclodextrin (HP-β-CD) and randomly methylated β-cyclodextrin (RAMEB), with RAMEB being more effective. The drug’s solubility varied across different pH buffers, being higher in acidic buffers. Interestingly, mefloquine’s solubility decreased with a citrate buffer, possibly due to precipitation. The NMR studies highlighted non-covalent interactions between RAMEB, HP-β-CD, and mefloquine, explaining the solubilizing effect via complexation phenomena. Furthermore, the NMR experiments indicated the complexation of mefloquine by all the studied cyclodextrins, forming diastereoisomeric complexes. Cyclodextrin complexation improved mefloquine’s solubility, potentially impacting its bioavailability.

## 1. Introduction

Malaria is an infectious disease transmitted by a parasite of the genus *Plasmodium* whose vector is a female mosquito of the genus *Anopheles*. In 2016, the World Health Organization (WHO) reported 216 million cases of malaria, 90% of which were in Africa. The child population (<5 years of age) is at high risk of contracting malaria, and 285,000 of the 445,000 individuals who died that year were children under 5 years of age. For the latter, mefloquine at a dose of 5 mg/kg/week can be used off-label for the prophylaxis of malaria and at a dose of 25 mg/kg (as a split doses) for the treatment of uncomplicated malaria [1]. Due to the lack of an age-appropriate formulation, mefloquine has been identified as a pediatric need by the European Medicine Agency and the U.S. Food and Drug Administration. It would, therefore, be pertinent to formulate an oral solution of mefloquine with a concentration between 10 and 20 mg/mL. This consideration is based on the required dosing regimen and the feasible volume of administration for the specific age group in question [2].

**Figure 1:**
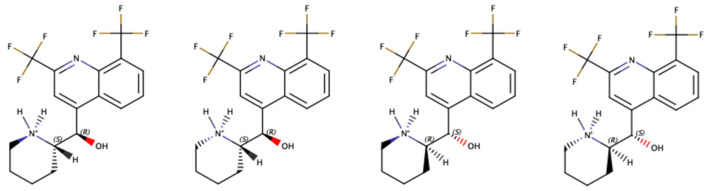
Chemical structure of the four diastereoisomers of mefloquine hydrochloride.

From a chemical and biopharmaceutical point of view, mefloquine is a 4-quinolinemethanol derivative that exists as a racemic mixture of (−)-(11*R*,12*S*)-*erythro*-mefloquine and (+)-(11*S*,12*R*)-*erythro*-mefloquine. The (+)-(11*S*,12*R*)-*erythro*-mefloquine enantiomer is a more potent antimalarial, whereas the (−)-(11*R*,12*S*)-*erythro*-mefloquine shows better binding capacities to the adenosine receptor which is believed to account for the neuropsychiatric side effects of mefloquine [3]. Because of piperidine’s basicity (pKa 8.6), mefloquine is a weak base that can form four diastereoisomers when protonated (Figure 1). It is commercialized as a hydrochloride salt, with reported solubilities of 1.950 mg/mL (acetate buffer, pH 4.5) and 1.806 mg/mL (water, pH 6.5) at 37 °C [4]. Recent caco-2 studies have highlighted a mid-permeability of mefloquine, measured around 9 × 10^−6^ cm/s, and moderate p-Gp inhibition [5]. Regarding these data, mefloquine may be classified as a class II (low solubility, high permeability) or a class IV (low solubility, low permeability) drug in the Biopharmaceutical Classification System [6] and as a class II drug (poorly soluble, extensively metabolized) in the Biopharmaceutics Drug Disposition Classification System [7].

Different approaches have been used to increase the aqueous solubility of mefloquine: optimization of the pH associated with co-crystallization [8], emulsification [9], encapsulation by liposomes [10], or complexation with β-cyclodextrin (β-CD) [11]. This latter compound belongs to the cyclodextrin family, which includes cyclic oligosaccharides composed of six, seven, or eight glycosidic units. Cyclodextrins have previously demonstrated their ability to form inclusion complexes with lipophilic molecules, especially those containing aromatic rings [12]. The formation of an inclusion complex between cyclodextrin and a guest molecule could enhance various properties of the guest molecule, such as its solubility, permeability, bioavailability, stability, and palatability, thereby potentially improving its therapeutic efficacy and patient acceptability [13,14,15]. This presents a valuable opportunity in the pharmaceutical formulation of mefloquine, a class II BCS drug in which, as Loftsson suggested, the use of cyclodextrins could notably enhance bioavailability [14]. The drug’s pronounced bitterness [16] and photolability [17] could also potentially be alleviated through the utilization of cyclodextrins.

The natural cyclodextrins have been chemically modified by replacing the hydroxyl groups at positions 2, 3, and 6 of the glycosidic units with hydroxypropyl-, methyl-, or sulfobutylether- groups with varying degrees of substitution, allowing for the adjustment of the solubility and interaction properties of cyclodextrins. For instance, the solubility of beta-cyclodextrin has been increased to more than 20 times its original value with sulfobutylether, hydroxypropyl, and randomly methylated beta cyclodextrin (RAMEB) derivatives [13]. These two last cyclodextrins, being commercially available in large quantities, led us to consider their potential benefits in formulating a mefloquine solution in line with Patil’s previous work [11].

The aim of this work is to determine the optimal cyclodextrin to use and the amount required to achieve a solubility of approximately 10 mg/mL of mefloquine in an oral solution. We focus on studying the complexation of mefloquine hydrochloride by modified β-cyclodextrins and assessing their impact on the solubility and stability of mefloquine.

## 2. Materials and Methods

### 2.1. Materials

Mefloquine hydrochloride (reference PHR1705) was purchased from Sigma Aldrich (Saint Louis, MO, USA). The (11*S*, 12*R*)-mefloquine and (11*R*, 12*S*)-mefloquine enantiomers were purified by the laboratory [2].

Cavasol W7 HP pharma hydroxypropyl-β-cyclodextrin (HP-β-CD) and Cavasol W7 M pharma randomly methylated β-cyclodextrin (RAMEB) were produced by Wacker Chemie AG (Munich, Germany) and purchased from Ashland (Covington, KY, USA). Heptakis(2,6-*O*-dimethyl)beta-cyclodextrin (DIMEB) was purchased from Cis BIO (Gif sur Yvette, France).

### 2.2. Solubility Studies

Mefloquine hydrochloride’s solubility was measured in triplicate within a sodium citrate buffer (50 mM, pH 3), a sodium phosphate buffer (80 mM, pH 2.7), a sodium phosphate buffer (50 mM, pH 4.6), a sodium phosphate buffer (50 mM, pH 6.6), and water for injection (WFI). For that purpose, an excess amount of mefloquine hydrochloride was added to 5 mL of the solutions to produce suspensions. These suspensions were sonicated for 1 h and stirred for 24 h at 24 °C and protected from the light. Aliquots were drawn from these suspensions and filtered through a Pall Life Science (Cortland, NY, USA) 0.45 µm filter with a Versapore acrylic copolymer membrane before quantification. The pH of these suspensions was checked before filtration.

Mefloquine phase solubility studies with cyclodextrins were carried out according to the Higuchi and Connors method [18] with a quantity of cyclodextrins not exceeding ten times the target concentration of mefloquine. Cyclodextrins solutions (from 26 mM to 264 mM) were prepared within sodium citrate (50 mM, pH 3) and sodium phosphate (80 mM, pH 2.7) buffer solutions and WFI. An excess amount of mefloquine was added to 5 mL of cyclodextrin solution in a volumetric flask to produce a suspension. The mefloquine suspensions were then sonicated for 1 h and stirred for 18 h at 24 °C and protected from the light. Aliquots were drawn from these suspensions and filtered through a Pall Life Science (Cortland, NY, USA) 0.45 µm filter with a Versapore acrylic copolymer membrane before quantification. The pH of these suspensions was checked before filtration.

### 2.3. Quantification of Mefloquine

Quantification of mefloquine was performed using a Shimadzu^®^ Nexera^®^ high-performance liquid chromatography system (Kyoto, Japan). One microliter of samples was eluted on a 150 mm × 2.1 mm 1.7 µm octadodecyl Acquity^®^ column (Waters, Milford, CT, USA) at 40 °C using an isocratic ammonia buffer(120 mM, pH 10):acetonitrile:methanol (40:40:20, *v*/*v*/*v*) mobile phase at a 1 mL/min flow rate. The samples were diluted to 1/10th with the mobile phase before injection. Quantification was performed at 283 nm with daily made calibration curves. This method was linear for concentrations ranging from 10 to 5000 mg/L. The limit of quantification was estimated at 48.44 mg/L and the repeatability and intermediate fidelity were good (coefficient of variation < 5%) for concentrations ranging from 50 to 5000 mg/L.

### 2.4. Complexation Efficiency

Complexation efficiencies (CE) were determined from the phase solubility studies using the Loftsson method [19].

CE = (D/CD)/(CD) = Slope/(1 − Slope)

where (D/CD) is the concentration of dissolved complex; (CD) is the concentration of dissolved free CD, and Slope is the slope of the phase-solubility profile.

### 2.5. Nuclear Magnetic Resonance Analysis of Complex Formation

Nuclear magnetic resonance (NMR) analyses were performed in a stepwise manner and one dimensional ^1^H-NMR experiments were performed first in order to detect interactions between compounds and determine if a complex could be formed. All ^1^H and ^19^F NMR experiments were recorded at the respective frequencies of 600.17 MHz and 564.72 MHz on a Bruker Avance III 600^®^ instrument (Wissembourg, France) equipped with a multinuclear z-gradient BBFO probe. The spectra were recorded at 300 K with a rigorous control of the temperature (±0.1 K). The internal calibration of the ^1^H NMR spectra was carried out using the residual HOD solvent signal at 4.71 ppm. The 90° pulse values were about 7.5 μs for the proton. The proton spectra were acquired with 128 scans and a 64 K time domain and, in ^19^F NMR, with 16 scans and a 32 K time domain, unless specified otherwise. A baseline correction was performed on each spectrum.

The stoichiometry of the complex was determined using Job’s method. Briefly, the Job plot procedure was applied: 11 solutions were prepared by mixing the mefloquine stock solution (5 mM in D_2_O) with the HP-β-CD or RAMEB stock solution (5 mM in D_2_O) at volumetric ratios ranging from 0 to 1. The total molar concentration and the total volume were kept constant at 5 mM and 500 µL, respectively, and the molar ratio of each component was varied from 0 to 1. Corresponding ^19^F NMR spectra were recorded. The observation of chemical shifts varying in a linear fashion with the concentrations of bound species was obtained and affords the corresponding Job plots as described elsewhere [18]. 

^19^F NMR Titrations: the same experimental data set of Job plot titrations was used. The association constants were obtained by fitting a 1:1 isotherm according to the programs available at http://app.supramolecular.org/bindfit/ (accessed on 19 October 2023) [20].

To complete these experiences, T-ROESY experiments were conducted to analyze dipolar interactions between mefloquine and cyclodextrins [21]. The T-ROESY experiments were recorded with 224 scans and a spectral window of 10 ppm in each of the dimensions (1024 real points in the F2 dimension and 256 points in the F1 dimension). The mixing time was set to 380 ms. COSY experiments were also performed to assign the dipolar interactions observed on the T-ROESY spectrum. Details concerning the experimental conditions are given in the figure captions. 

### 2.6. Photostability Studies

The stability of the RAMEB complex was examined and compared to a solution of mefloquine hydrochloride solubilised with a 50:50 mixture of acetonitrile and water as recommended by the International Conference on Harmonisation (ICH) standards to poorly soluble compounds [22], both at an equivalent concentration of 10 mg/mL. The solutions were stored in type 1 glass vials (n = 3 for each solution) sealed with a rubber septum. To solubilize mefloquine hydrochloride in this experiment, 200 mM of RAMEB was used. Photostability studies were conducted using the Binder KBF P240 climatic chamber (Tuttligen, Germany), equipped with UVA and visible illumination sources set at 7500 Lux and 1.1 W/m^2^. The chamber was maintained at a temperature of 25 ± 2 °C and a relative humidity of 60 ± 5% during the 6-months study.

The HPLC method outlined in the European Pharmacopoeia for the assessment of related substances of mefloquine was modified to quantify mefloquine and its potential degradation products. The method was adapted using pharmacopeia recommendations for utilization with the Phenomenex^®^ Kinetex^®^ 2.6 µm, 150 × 2.1 mm column (Torrance, CA, USA), featuring the same octadecyl stationary phase with a smaller particle size. By employing this column, the flow rate was optimized at 0.4 mL/min.

## 3. Results and Discussion

### 3.1. Analysis of Mefloquine Solubilization with Hydroxypropyl-β-Cyclodextrin (HP-β-CD) and Randomly Methylated β-Cyclodextrin (RAMEB)

The solubility of mefloquine was measured at 4.12 mg/mL in purified water, resulting in a final pH of 4.6 at 24 °C. As expected, the solubility of mefloquine was higher in the acidic buffers compared to the neutral ones. It ranged from 4.81 mg/mL in a phosphate buffer at a pH of 2.6 to 0.78 mg/mL in a phosphate buffer at a pH of 6.5. An exception was observed with a citrate buffer at a pH of 2.7, in which the solubility was measured at 0.64 mg/mL.

The results show a two-fold higher solubility of mefloquine hydrochloride than previously reported [4]; this may be related to the fact that there are at least five polymorphic forms of mefloquine hydrochloride and that polymorphism may be associated with different physico-chemical properties [23]. Our results also show that buffer species may account for different solubility at the same pH value, with the citrate buffer producing an eight-fold decrease in the solubility of mefloquine compared to the phosphate buffer. Although not studied, precipitation of mefloquine may occur with sodium citrate due to common ion effects.

**Figure 2:**
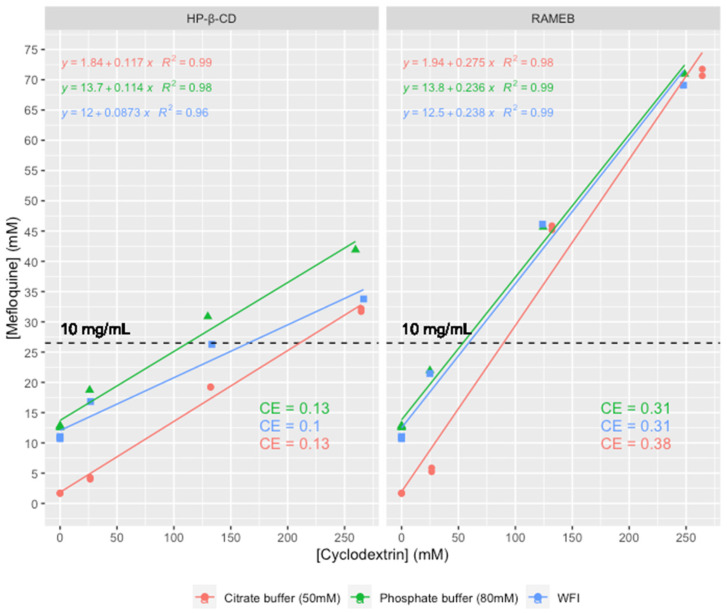
Phase diagrams obtained for mefloquine in presence of hydroxypropyl-β-cyclodextrin (HP-β-CD) or randomly methylated β-cyclodextrin (RAMEB) using a citrate buffer (pH 2.7–2.9), a phosphate buffer (pH 2.6–2.8), or water for injection (WFI, pH 4.3–4.6), with the linear regression equation, correlation coefficient, and complexation efficiency (CE) depicted on it. The target of 10 mg/mL mefloquine content has been depicted.

The solubility of mefloquine was enhanced with the HP-β-CD or RAMEB solutions (Figure 2).

As found previously with β-CD, HP-β-CD and RAMEB linearly increase the solubility of mefloquine suggesting an A_L_ phase solubility profile [11]. Moreover, it is often assumed that the linear trend of the phase solubility diagram indicates the formation of a 1:1 complex. For a guest molecule with limited aqueous solubility, it is well-known that the association’s constant value cannot be determined accurately with this method, and the notion of complexation efficiency (CE) was introduced by Loftsson et al. to compare the solubilizing effect of different CDs on a guest molecule [19].

The initial solubility of mefloquine in the phosphate buffer (pH 2.9, 24 °C) increased by factors of four and nine upon the addition of 264 mM of HP-β-CD and RAMEB, respectively. The latter was more effective, achieving the target concentration of 26.5 mM (10 mg/mL) with approximately two equivalents of RAMEB. These results can be compared to the previously reported increase in solubility of 1.18 in the presence of β-CD [11]. The decrease in mefloquine solubility observed for the citrate buffer (50 mM, pH 2.9) was also observed in the presence of cyclodextrins. The drug/cyclodextrin/hydroxy acid multicomponent systems were not synergistic, and these results corroborate those previously reported regarding the requirements for exploiting the properties of multicomponent complexation (i.e., drug solubility should be low and should be improved by salt formation with hydroxy acids) [24].

The complexation efficiencies (CE) of mefloquine with HP-β-CD and RAMEB were calculated based on the slope of the solubility curves, which were 0.1–0.13 for HP-β-CD and 0.31–0.38 for RAMEB, respectively. They were not very sensitive to the solution medium.

### 3.2. Nuclear Magnetic Resonance Analysis of Mefloquine–CD Interaction

Nuclear magnetic resonance (NMR) is a powerful technique to probe supramolecular interactions in solution between CDs and small molecules. In this context, it was used to highlight potential interactions between mefloquine and CDs that could explain the solubilizing effect of CDs towards mefloquine via complexation phenomena.

**Figure 3:**
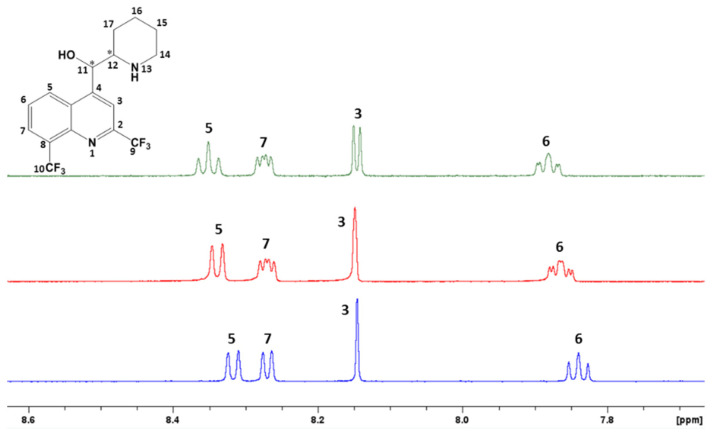
Partial ^1^H NMR spectrum in the aromatic area (600 MHz, 300 K) of mefloquine (5 mM in D_2_O) alone (**bottom**) and in the presence of HP-β-CD (5 mM in D_2_O) (**middle**) and of RAMEB (5 mM in D_2_O) (**top**).

Firstly, ^1^H NMR experiments of mefloquine alone and in the presence of RAMEB or HP-β-CD were performed. For CDs–mefloquine mixtures, chemical shift variations were observed both for mefloquine, with a de-shielding of most aromatic protons (Figure 3), and for broad ^1^H signals of RAMEB and HP-β-CD when compared to the compounds alone.

**Figure 4:**
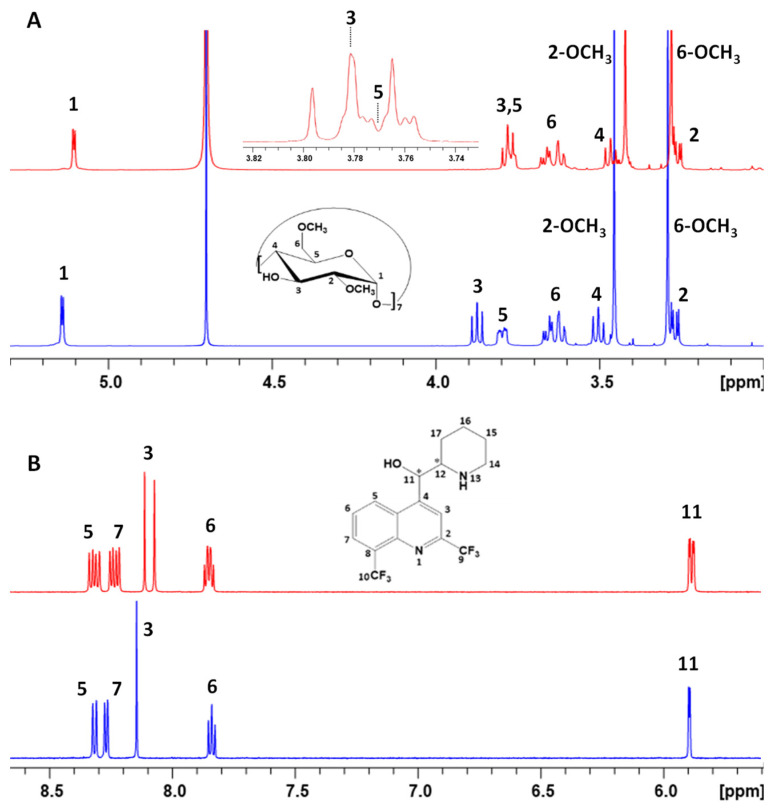
(**A**) Partial ^1^H NMR spectrum (600 MHz, 300 K) of DIMEB alone (5 mM in D_2_O) (**bottom**) and in the presence of mefloquine (5 mM in D_2_O) (**top**). (**B**) Partial ^1^H NMR spectrum showing the aromatic protons and the proton H11 (600 MHz, 300 K) of mefloquine when alone (5 mM in D_2_O) (**bottom**) and in the presence of DIMEB (5 mM in D_2_O) (**top**).

These observations agree with the non-covalent interactions occurring between RAMEB, HP-β-CD, and mefloquine. However, as already demonstrated [25,26], it was not simple for RAMEB and HP-β-CD, due to their multi-component character with a tremendous number of isomers and their broad ^1^H signals, to determine accurately which protons were involved in these variations of chemical shifts. Only for RAMEB, the corresponding pure heptakis-2,6 di-*O*-methylated β-CD called DIMEB could be used as a model to go deeper in our NMR investigation later. For the DIMEB–mefloquine mixture and compared to DIMEB alone, a very important chemical shift variation for the proton H-3 of DIMEB and a weaker one for H-5 were observed for these two protons located inside the DIMEB cavity supporting the formation of an inclusion complex (Figure 4A). It should be noted that, in our hands, H-3 and H-5 signals are superimposed despite different NMR spectrum processing parameters. But, important chemical shift variations were also observed for DIMEB protons H-1, H-4, H-2, and 2-OCH_3,_ revealing that non-covalent interactions of mefloquine with the outside of DIMEB could not be excluded at this stage. An important shielding of the aromatic protons H7 and H3 in particular was also observed on the ^1^H NMR spectrum of mefloquine in the presence of DIMEB when compared to mefloquine alone (Figure 4B).

Moreover, another evidence of complexation of mefloquine by all the studied CDs was the splitting observed in ^1^H NMR for every aromatic signal and the proton H11 of mefloquine (Figure 3 and Figure 4B). Indeed, mefloquine exists under a racemic mixture, and the doubling of signals can be explained with the chiral differentiation of each enantiomer with CDs through the forming diastereoisomeric complexes.

**Figure 5:**
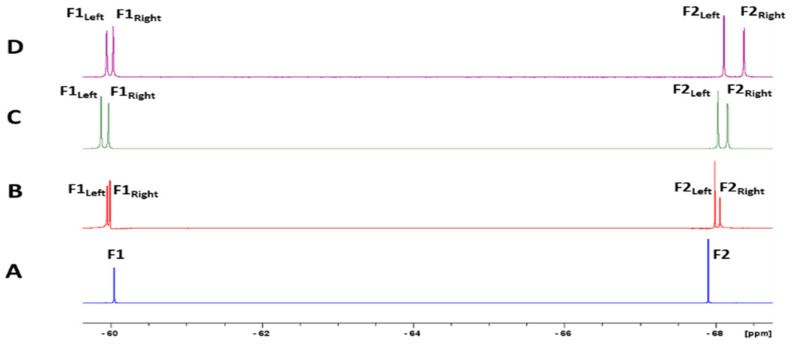
Partial ^19^F NMR spectrum (564 MHz, 300 K) of mefloquine alone (5 mM in D_2_O) (**A**) and in the presence of β-CD (5 mM in D_2_O) (**B**), of RAMEB (5 mM in D_2_O) (**C**), and of DIMEB (5 mM in D_2_O) (**D**). F1: the most de-shielded ^19^F signal; F2: the most shielded ^19^F signal. After splitting, F1_Left_/F2_Left_ and F1_Right_/F2_Right_ correspond to leftmost (more de-shielded) and rightmost (more shielded) ^19^F signals of each signal, respectively.

With ^19^F being an NMR-sensitive nucleus and mefloquine bearing two CF_3_ groups on its structure, a direct ^19^F NMR experiment was considered next for mefloquine alone and in a mixture with CDs to explore the ^19^F signals’ behaviour towards complexation. The ^19^F NMR spectrum of mefloquine alone showed two singlets at around −60 and −68 ppm, corresponding to the two CF_3_ groups we named F1 and F2, respectively. In the presence of HP-β-CD, RAMEB, and DIMEB, chemical shifts variations and a splitting more or less important of the ^19^F signals of mefloquine were observed, as previously, due to the chiral differentiation of both enantiomers (F1 → F1_Left_ + F1_Right_, F2 → F2_Left_ + F2_Right_) under complexation. We noticed that the ^19^F signals of mefloquine did not evolve in the same way, namely that the fluorine F1 signal at −60 ppm was downfield-shifted, while the signal F2 at −68 ppm was upfield-shifted (Figure 5).

To assign the ^19^F signals of each mefloquine enantiomer in the presence of CDs, the purified mefloquine enantiomer (11*R*, 12*S*) chemically synthetized in our lab was added to create an enantiomeric excess and increase the corresponding ^19^F signals compared to the other enantiomer (11*S*, 12*R*). Thus, the F1_Left_ and F2_Right_ signals were attributed to (11*S*, 12*R*)-mefloquine, whereas the F1_Right_ and F2_Left_ signals were attributed to (11*R*, 12*S*)-mefloquine. The same approach was also applied in ^1^H NMR to assign the signals of each enantiomer.

To gain insight on mefloquine–DIMEB interactions, a 2D ^1^H-^1^H T-ROESY experiment was performed to highlight the spatial proximity occurring between nuclei via dipolar couplings. The presence of cross-correlation peaks between the protons of mefloquine and those of DIMEB confirmed the intermolecular interaction between the two partners. Unfortunately, a very small difference in chemical shift was observed between the H-3 and H-5 of DIMEB (0.01 ppm, Figure 4A) due to the formation of inclusion complexes preventing the cross-correlation peaks involving these protons on the T-ROESY to be unambiguously differentiated. 

**Figure 6:**
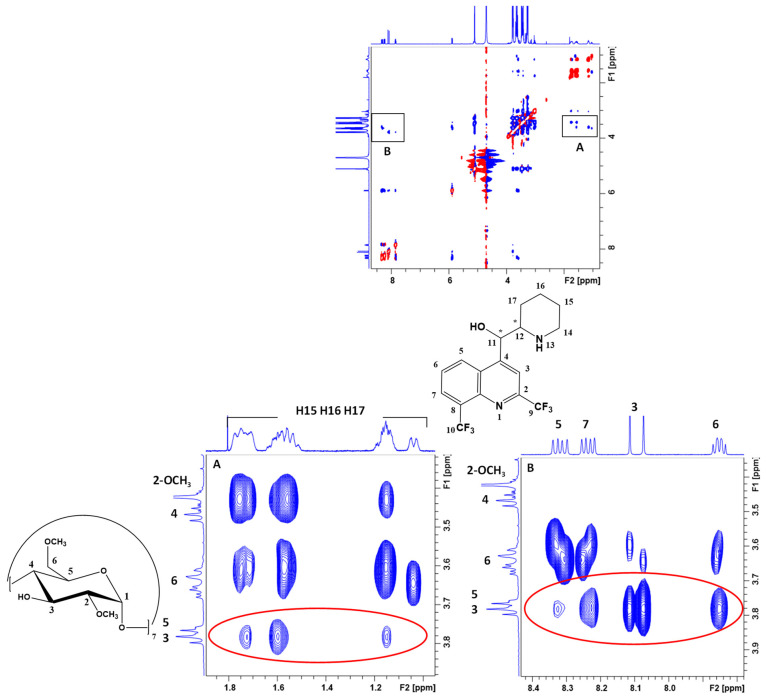
T-ROESY spectrum (600 MHz, 300 K) of mefloquine (5 mM in D_2_O) in the presence of DIMEB (5 mM in D_2_O) (**top**). Expansion of the piperidine (**A**, **left**) and quinolein area inset on the T-ROESY spectrum (**B**, **right**). Intermolecular interactions between DIMEB and mefloquine are indicated with red circles; other cross-peaks correspond to the intramolecular ROE of mefloquine.

On the T-ROESY spectrum (Figure 6), intense cross-peaks of intramolecular interactions of mefloquine were observed between nearby protons, which had also been observed during the COSY experiment (Figure 6A), or between the piperidine moiety and the benzenic ring of quinolein (Figure 6B). Regarding intermolecular interactions with the DIMEB cavity (H-3 and/or H-5), the most intense cross-peaks involved H3 and H6 of mefloquine (both sides of quinolein). The weakest interactions involving H7 (quinolein) and the protons of piperidine (H15, H16, H17) with the H-3 and/or H-5 of DIMEB were also observed. Despite the lack of resolution to clearly separate the H-3 and H-5 proton signals from DIMEB, it could be noted that cross-peaks between mefloquine and DIMEB are mainly centered in the middle of H-3, allowing us to suppose that inclusion might happen via the secondary face. This observation is supported by the absence of dipolar interactions between mefloquine and the protons H-6 or 6-OCH_3_ of DIMEB.

Taking into account all this information, DIMEB could interact mainly on its wider side with the quinolein part (benzenic and pyridin rings) and piperidine part of mefloquine. To conclude, several mefloquine–DIMEB inclusion complexes seem to co-exist in solution. Moreover, the T-ROESY experiment did not reveal discriminant cross-peaks between both the enantiomers of mefloquine and DIMEB that could have shown different modes of interaction for each enantiomer of mefloquine with DIMEB. To complete the characterization of mefloquine–CDs complexes, the stoichiometry and average association constants for RAMEB and HP-β-CD were attempted to be determined with a graphical approach using the Job plot and titration methods, respectively.

**Figure 7:**
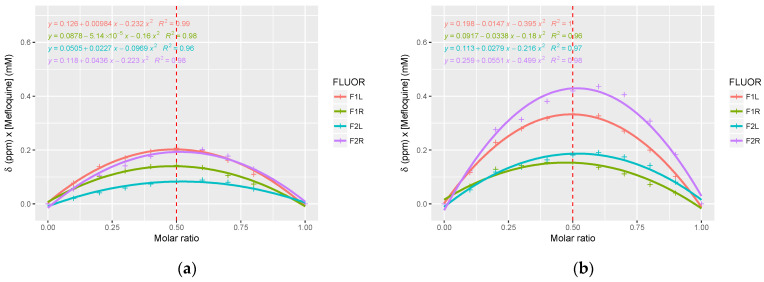
The Job plots (^19^F NMR spectrum) obtained (**a**) with HP-*β*-CD and (**b**) with RAMEB. The F1_Left_ and F2_Right_ signals were attributed to (11*S*, 12*R*)-mefloquine, whereas the F1_Right_ and F2_Left_ signals were attributed to (11*R*, 12*S*)-mefloquine. Quadratic regression curves and the corresponding coefficients of correlation were depicted on the figures, and a red dotted line was plotted to show the molar ratio corresponding to a 1:1 complex stoichiometry.

The Job plots were obtained using ^19^F NMR rather than ^1^H NMR, since it made it easier to monitor chemical shift variations and enables us to overcome the signals’ overlapping often encountered in ^1^H NMR (Figure 7). Although not perfectly symmetrical, the curves were obtained with a maximum around 0.5 supporting the formation of a mainly 1:1 complex for RAMEB and HP-β-CD. 

**Figure 8:**
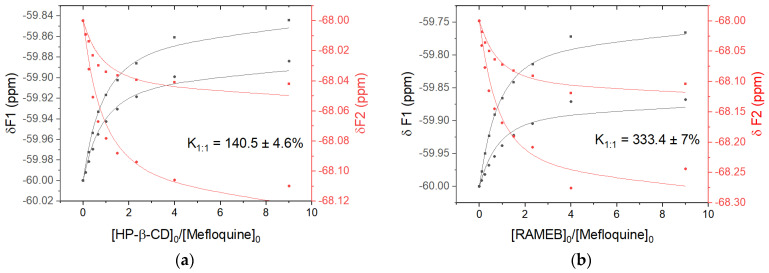
^19^F NMR titration 1:1 isotherms created by monitoring variations in the chemical shift of F-1L, F-1R, F-2L, and F-2R as a function of [HP-β-CD]_0_/[mefloquine]_0_ (**a**) and [RAMEB]_0_/[mefloquine]_0_ (**b**) ratio.

The binding affinities between mefloquine and both cyclodextrins were evidenced by a ^19^F NMR titration (Figure 8). For the sake of clarity, the same experimental data set of Job plot titrations was used. 

By tracking the change in the chemical shifts of the four ^19^F signals, the average associate constants (K1:1) with HP-β-CD and RAMEB were determined to be 140.5 M^−1^ and 333.4 M^−1^, respectively. They were found in agreement with the CE measured with a phase solubility diagram and the value reported in the literature for β-CD (120 M^−1^) [11]. However, it should be noted that the 1:1 model does not perfectly fit the data used, which may be explained by the presence of more than one equilibrium, as suggested by the use of CD mixtures, the slightly asymmetric Job plots, and the T-ROESY experiments described above.

### 3.3. Analysis of the Photostability of Mefloquine in the Presence of CD

**Figure 9:**
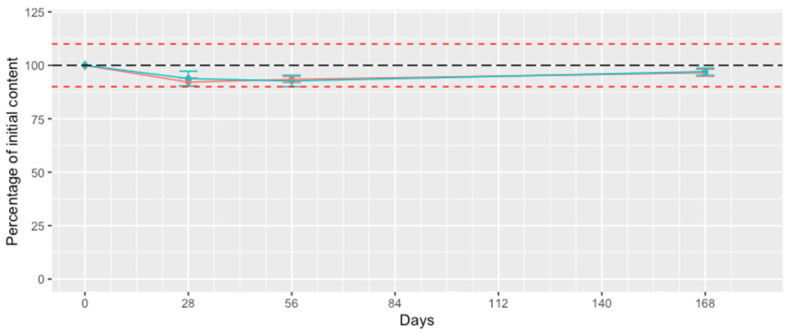
Variation in mefloquine content (mean ± sd, n = 3) within the cyclodextrin solution (blue line) and the acetonitrile solution (red line) over time. Red dashed lines are placed at 90 and 110% of the initial content (black long dashed line).

The aim of this study was to determine whether the presence of the most promising cyclodextrin affects the stability of mefloquine in an aqueous solution. No color change was been observed for the RAMEB solutions and the acetonitrile:water (50:50) solutions. No substantial change in mefloquine content was observed over the 168 days of the study (Figure 9). This absence of change in mefloquine content was further supported by the analysis of the chromatograms, which did not reveal any additional peak appearances. Therefore, it seems reasonable to conclude that the use of RAMEB has no negative or positive influence on the stability of mefloquine, which appeared to be stable in an aqueous medium and when exposed to light during this period, despite a previous study indicating that, under conditions of photodegradation exceeding the scope of the ICH guidelines, mefloquine could generate degradation products [17]. Since the improvement in stability for certain active substances through complexation with cyclodextrins has already been described [27], as well as the catalysis of degradation reactions of guest molecules [28], it seems reasonable to systematically examine the influence of cyclodextrins on the stability of a guest molecule.

## 4. Conclusions

The solubility of mefloquine is significantly influenced by pH, exhibiting greater solubility in acidic conditions. However, the solubility also depends on the buffer species, with an eight-fold decrease in solubility noted in a citrate buffer compared to a phosphate buffer at the same pH. These observations suggest the importance of pH and buffer choice when considering the solubilization and delivery of mefloquine. 

The solubility of mefloquine is significantly enhanced by complexation with hydroxypropyl-β-cyclodextrin (HP-β-CD) and randomly methylated β-cyclodextrin (RAMEB), and this enhancement was observed across the varying pH levels tested. This effect is attributed to the formation of an inclusion complex between these cyclodextrins and mefloquine, with RAMEB showing a greater efficiency.

These findings highlight the potential of cyclodextrin complexation as a promising strategy for improving the solubility and, potentially, the bioavailability of mefloquine, which could have critical implications for its use in malaria prophylaxis and treatment in children.

## Data Availability

Data are contained within the article.
